# Advancements in the Growth and Construction of Recombinant Lumpy Skin Disease Virus (LSDV) for Use as a Vaccine Vector

**DOI:** 10.3390/vaccines9101131

**Published:** 2021-10-04

**Authors:** Michiel van Diepen, Rosamund Chapman, Nicola Douglass, Leah Whittle, Nicole Chineka, Shireen Galant, Christian Cotchobos, Akiko Suzuki, Anna-Lise Williamson

**Affiliations:** 1 Department of Pathology, Division of Medical Virology, Faculty of Health Sciences, University of Cape Town, Cape Town 7925, South Africa; michiel.vandiepen@uct.ac.za (M.v.D.); ros.chapman@uct.ac.za (R.C.); whtlea002@myuct.ac.za (L.W.); chnane006@myuct.ac.za (N.C.); shireen.galant@uct.ac.za (S.G.); ctcchr001@myuct.ac.za (C.C.); SZKAKI001@myuct.ac.za (A.S.); 2 Institute of Infectious Disease and Molecular Medicine, University of Cape Town, Cape Town 7925, South Africa

**Keywords:** lumpy skin disease virus, vaccine vector, BHK-21 cells, RK13 cells, K1L host range

## Abstract

Attenuated vaccine strains of lumpy skin disease virus (LSDV) have become increasingly popular as recombinant vaccine vectors, to target both LSDV, as well as other pathogens, including human infectious agents. Historically, these vaccine strains and recombinants were generated in primary (lamb) testis (LT) cells, Madin–Darby bovine kidney (MDBK) cells or in eggs. Growth in eggs is a laborious process, the use of primary cells has the potential to introduce pathogens and MDBK cells are known to harbor bovine viral diarrhea virus (BVDV). In this study, data is presented to show the growth of an attenuated LSDV strain in baby hamster kidney (BHK-21) cells. Subsequently, a recombinant LSDV vaccine was generated in BHK-21 cells. Partial growth was also observed in rabbit kidney cells (RK13), but only when the vaccinia virus host range gene K1L was expressed. Despite the limited growth, the expression of K1L was enough to serve as a positive selection marker for the generation of recombinant LSDV vaccines in RK13 cells. The simplification of generating (recombinant) LSDV vaccines shown here should increase the interest for this platform for future livestock vaccine development and, with BHK-21 cells approved for current good manufacturing practice, this can be expanded to human vaccines as well.

## 1. Introduction

Poxviruses have a long history of being used as vectors for recombinant vaccines [1,2]. Most of the registered recombinant poxvirus vaccines are for veterinary use and include the canarypox virus (CNPV) based vector ALVAC for diseases such as rabies, feline leukaemia virus and equine influenza [3,4,5]. Due to their safety profiles, the majority of poxviruses being explored for human use do not complete their replication cycle in humans and include canarypox virus, various vaccinia virus strains and lumpy skin disease virus (LSDV) [6,7,8,9,10]. Recently, the first human vaccine based on a recombinant poxvirus was approved by regulatory bodies for Ebola [11]. This vaccine is based on modified vaccinia Ankara (MVA), which does not complete its replication cycle in human cells. The vaccine encodes glycoproteins of Ebola virus Zaire, Sudan virus and Marburg virus and the nucleoprotein of the Thai Forest virus. It is given as a boost in a heterologous Ebola vaccination regimen with an Adenovirus vectored priming vaccine. 

Combinations of different poxvirus vectored vaccines can give different types of immune response. In rhesus macaques, there were different systemic proinflammatory and antiviral cytokine and chemokine levels following vaccination with the canarypox virus vector, ALVAC, compared to the vaccinia virus (VACV)-derived vectors MVA and NYVAC [12]. Rhesus macaques, vaccinated in a heterologous prime boost regimen consisting of a recombinant modified vaccinia virus Ankara (rMVA) prime/recombinant fowlpox virus (rFPV) boost or recombinant vaccinia virus prime/rFPV boost, developed comparable cellular immune responses, which were greater in magnitude than those developed in animals that received homologous prime/boost with rMVA [13]. On testing candidate HIV-1 vaccines, heterologous prime boost with rMVA and rLSDV expressing HIV-1 antigens gave a better T cell response than either viral vector alone [9].

LSDV is a member of the *Capripoxvirinae* genus of the *Poxviridae* family [14] and has a host range restricted to ruminants. LSDV causes serious disease in cattle and there are effective attenuated and inactivated vaccines available [15,16]. Recombinant LSDV vaccines that have been tested in cattle or sheep include dual vaccines against LSDV + rabies [17] and LSDV + rift valley fever virus [18]. Pre-clinical studies of LSDV as an HIV vaccine have also taken place [9,10]. The promising immune responses to LSDV in replication-restricted hosts have supported the development of LSDV as a vaccine for humans [19,20]. However, the present manufacture of vaccines takes place in cell lines or primary cultures [21,22], which are not suitable for the manufacturing of human vaccines according to current good manufacturing practice. Madin–Darby bovine kidney (MDBK) cells can be used for culture in veterinary use, but this cell line is often contaminated with bovine viral diarrhea virus (BVDV). Passaging through embryonated chicken eggs can be used to remove this BVDV [23], but eggs are not considered a viable alternative for manufacture of vaccine. Recently, it was reported that Capripoxviruses grow well in cultures of the embryonic skin of sheep (ESH-L) and primary foetal heart cells, but to a lesser extent in Vero cells and an ovine testis cell line [21]. One of the aims of our study was to determine if the manufacture of LSDV could be done in a baby hamster kidney cell line (BHK-21), which is suitable for vaccine manufacture for human vaccines. An investigation was made into the construction of recombinant LSDV in BHK-21 cells. Based on the function of VACV C6 protein as a multifunctional interferon (IFN) antagonist responsible for proteasomal degradation of class II histone deacetylase 4 and 5 (HDAC4, HDAC5), with both HDACs inhibiting VACV replication in vitro [24,25], it was hypothesized that the HDAC inhibitor, sodium butyrate, could impact on the growth of LSDV in BHK-21 cells. 

Host range genes have implications for both growing poxviruses in vitro and for generating recombinant vaccines. The K1L gene, when recombined into MVA, enabled the rMVA to grow in cell lines outside of its normal host range [26,27]. K1L has been shown to inhibit Nuclear Factor kappa B (NF-kB) by preventing the degradation of IkBα, a known inhibitor of NF-kB [28,29]. NF-kB is a small group of inducible transcription factors that regulate DNA transcription, cytokine production, and cellular survival. Inhibition of NF-kB thus results in impaired proinflammatory gene expression by the host [30]. The inclusion of the K1L gene into transfer vectors has likewise facilitated the selection of recombinant MVA, as recombinants expressing K1L grown in rabbit kidney (RK) 13 cells, whilst the wild-type MVA does not [27]. Additionally, following transfection with K1L and antibiotic selection to generate a stable cell line expressing K1L, RK13 cells were seen to be permissive to MVA [31]. Similar to MVA, LSDV lacks the K1L gene [32] and is thus incapable of replicating in RK13 cells. One of the aims of this research was to evaluate the growth of LSDV in the presence of K1L in the RK13 cell line, which is not normally permissive to LSDV. 

The purpose of this study was to investigate ways to improve the growth and selection of recombinants of LSDV for both human and veterinary applications. LSDV was shown to grow in BHK-21 cells and K1L was shown to rescue growth of LSDV in RK13 cells.

## 2. Materials and Methods

### 2.1. Antibodies, Plasmids, Cell Lines and Reagents

Goat anti-HIV-1 gp160 (MRC ADP 72 408/5104), rabbit anti-HIV-1 p24 (Gag) (ARP 432), mouse mAb THE™ DYKDDDDK Tag Antibody for detection of the FLAG-tag (GenScript, Piscataway, New Jersey, United States, A00187), donkey anti-goat IgG FITC, donkey anti-rabbit IgG FITC or Cy3 and donkey anti-mouse IgG FITC or Cy3 (all Life Technologies, Carlsbad, California, USA) were used for immunofluorescence assays. Goat anti-HIV-1 gp120 (BioRad, Hercules, California, United States 5000-0557), goat anti-HIV-1 p24 (Gag) (BioRad 4999-9007), THE™ DYKDDDDK Tag Antibody, mouse monoclonal anti-goat/sheep IgG–AP GT34 (Sigma, St. Louis, Missouri, United States) and Goat Anti-Mouse IgG Antibody (H&L) [Alkaline phosphatase] (GenScript, Piscataway, New Jersey, United States, A10097) were used for western blotting. MDBK, RK13, HEK293T and BHK-21 cell lines were obtained from the American Type Culture Collection (ATCC, Manassas, Virginia, United States). All cells were grown in Dulbecco’s Modified Eagle’s Medium (DMEM) High Glucose + L-Glutamine + 10% Fetal Calf Serum (FCS) + 1x Pen/Strep (all Gibco, Carlsbad, California, United States). For serum-free media, no FCS was added. Recombinant LSDV was titrated in MDBK cells by counting fluorescent foci three days post infection to determine fluorescent forming units/mL (FFU/mL). 

The mammalian expression plasmid pTHpCapR [33] was used as a backbone for mammalian expression plasmids and pUC57simple (GenScript) was used as a backbone for pox transfer vectors. The recombinant nLSDVSODis-UCT and modified vaccinia Ankara (MVA) that expresses HIV-1 CAP256 Env + subtype C mosaic Gag (MVAGC5) have been previously described [34,35]. The construction of LSDVGC5 is described by Chapman et al. (in the concurrent special issue of Vaccines). LSDVGC5 expresses HIV-1 CAP256 Env + subtype C mosaic Gag [35].

All imaging was performed on a Zeiss Axio Microscope (Carl Zeiss AG, Oberkochen, Germany) and analysed with Zeiss Zen software (https://www.zeiss.com/microscopy/int/products/microscope-software/zen-lite.html (accessed on 4 October 2021)).

All graphs were plotted in GraphPad Prism 5.0 (GraphPad Software, San Diego, California, United States).

### 2.2. Promoter Activity in LSDV

Five promoters were tested for recognition by LSDV. These are shown in Table 1 and include a synthetic early-late promoter of VACV (pSS) [36], a synthetic early-late optimised promoter of VACV (pLEO)[37], a modified early fowlpox virus promoter (pmFP), which had the late portion of the promoter removed [38,39], a promoter of the 7.5 kilo Dalton (kDa) polypeptide gene of VACV (p7.5) [40] and a modified early-late promoter of the H5 gene of VACV (pmH5) [41]. Each promoter was cloned upstream of eGFP in pUC57simple and tested for transient expression, after transfection of BHK-21 cells, which were infected with nLSDVSODis-UCT. A 70% confluent layer of BHK-21 cells, in 12-well plates, were infected with LSDV (MOI = 0.5) and 2 h later transfected with 2 µg of the respective VACV promoter-eGFP plasmids using 1 µL of X-tremeGENE HP ( Roche, Basel, Switzerland). The eGFP signal was imaged as a proxy for VACV promoter activity two days after transfection. 

### 2.3. Generation of Recombinant LSDVGC5 Virus in BHK-21 Cells

A recombinant nLSDVSODis-UCT virus (LSDV(SODis)BEFV-Gb) containing an eGFP marker in the 49–50 locus (Douglass et al., concurrent special issue of Vaccines) was used to target the expression cassette of pmH5-Env, pLEO-mosaic Gag and pmFP-mCherry into the 49–50 locus, with a positive integration event marked by replacing eGFP expression with that of mCherry. A similar strategy was employed for the construction of LSDVGC5 described by Chapman et al. (in concurrent special issue of Vaccines).

BHK-21 cells were plated and infected at the same time with 1 µL LSDV(SODis)BEFV-Gb (1.8 × 10^7^ TCID_50_/_mL_). Two hours later, infected cells were transfected with 1 µg of the transfer vector pFLEx(49–50) CAP256 gp150-FL-IP Gag^M^ mCherry, using 1 µL of X-tremeGENE HP. Cells were frozen three days post transfection for passage 0 (P0). Single clones were generated in BHK-21 cells by either picking mCherry positive foci (P1) or by limited dilution ranges of cell lysate after freeze thawing (>P1). By P4, wells containing only mCherry positive recombinant LSDV were obtained, thus generating LSDVGC5 (BHK-21). Single foci were bulked up in T75, T175 and Hyperflasks using BHK-21 cells. After freeze-thawing, the virus was pelleted on a 36% sucrose cushion, reconstituted in PBS + 10% glycerol and stored at −80 °C. Correct integration into nLSDV(SODis)BEFV-Gb was verified by PCR, and expression of Env and Gag from LSDVGC5 (BHK-21) was assessed by western blotting and immunofluorescence. 

### 2.4. Growth Curve of LSDVGC5 in BHK-21 Cells

BHK-21 cells were infected with LSDVGC5 at MOI = 0.05 in triplicate (*n* = 3) with or without the pan-HDAC inhibitor 2 mM sodium butyrate (Sigma) in 12-well plates (*n* = 10 plates). As a control LSDVGC5 was added to three wells without cells in each plate. Cells were plated and infected on the same day. Wells were imaged daily and one plate was frozen each day for downstream titration after two freeze-thaw cycles to determine FFU/mL. This data was Log10 converted, plotted and analysed in GraphPad Prism 5.0.

### 2.5. Transgene Expression from MVAGC5 and LSDVGC5 in Non-Permissive Cells Stimulated with the pan-HDAC Inhibitor Sodium Butyrate

HEK293T cells were infected with MVAGC5 or LSDVGC5 at MOI = 0.5 in triplicate (*n* = 3) with or without the pan-HDAC inhibitor 2 mM sodium butyrate in 12-well plates (*n* = 4 plates). Cells were plated and infected on the same day. After three days, media was removed from one plate and cells were lysed with 500 µL Glo Lysis buffer (Promega, Madison, Wisconsin, United States). Equal volumes of cell lysate were run on protein SDS PAGE and expression of the MVAGC5 and LSDVGC5 transgene HIV-1 Env was confirmed by western blot analysis, imaged on a BioRad GelDoc XR, with densitometry performed with the accompanying ImageLab software (BioRad, Hercules, California, United States).

### 2.6. Generation of RK13 Cells with Stable Expression of VACV K1L

The VACV host-range gene K1L was PCR cloned into pTHpCapR to include a short C-terminal linker (GGGGS) and FLAG-tag (DYKDDDDK) upstream of the STOP codon, thereby generating the plasmid pMEx K1L-FLAG. The K1L gene was verified by DNA sequencing. Subsequently, an IRES-Neomycin resistance cassette was introduced directly downstream of K1L-FLAG to generate pMEx K1L-FLAG Ires Neo(r) (pMEx K1L-FLAG IN), which was used to make cell lines stably expressing K1L-FLAG. Transient expression in RK13 cells of K1L-FLAG from pMEx K1L-FLAG and pMEx K1L-FLAG-IN was verified by western blot analysis and immunofluorescence using the FLAG-tag. RK13 cells with stable expression of K1L-FLAG were generated by transfecting with pMEx K1L-FLAG-IN and passaging cells for >10 passages in media + 0.5 mg/mL Geneticin (Gibco). For this cell line, RK13 K1L, expression of K1L-FLAG was verified by western blot analysis and immunofluorescence. A subsequent clonal cell line, with detectable expression of K1L in all cells, as assessed by immunofluorescence, was identified by screening single clones from limited dilution ranges of RK13 K1L, thus generating RK13-K1L(H10).

### 2.7. LSDVGC5 Growth in RK13 Cells

LSDV titration experiments were performed in the RK13-K1L(H10) cell line. Cells were infected with LSDVGC5 (MOI = 0.3) (*n* = 3) and, after three days, virus was harvested by freeze-thawing and titrated in MDBK cells.

### 2.8. Generation of LSDV-K1L-eGFP in RK13 Cells

A recombinant LSDV, LSDV(SODis)BEFV-Ga, containing a mCherry marker in the 49–50 locus (Douglass et al., concurrent special issue of vaccines) was used to target a construct containing the VACV host-range gene K1L under its native promoter and pSS-eGFP into the 49–50 locus, with a positive integration event marked by replacing mCherry expression with eGFP. In this case, one day after plating, primary LT cells were infected with LSDV(SODis)BEFV-Ga and two hours later transfected with the transfer vector pLSDV-K1L-eGFP (5 µg, linearised with XhoI and BamHI, followed by heat inactivation) using 3µL X-tremeGENE HP. Cells were frozen two days post transfection for P0. Viral lysates were generated by freeze-thawing and used for passaging in RK13 cells. From P2 onwards, mCherry and eGFP positive foci in RK13 cells were counted. By P4, wells containing only eGFP positive recombinant LSDV were observed. Correct integration into LSDV(SODis)BEFV-Ga to produce LSDV-K1L-eGFP was verified by PCR. Clonal LSDV-K1L-eGFP generated in RK13 cells was further bulked-up in BHK-21 cells. 

## 3. Results

### 3.1. Expression of Foreign Genes by LSDV from Different Promoters 

**Figure 1:**
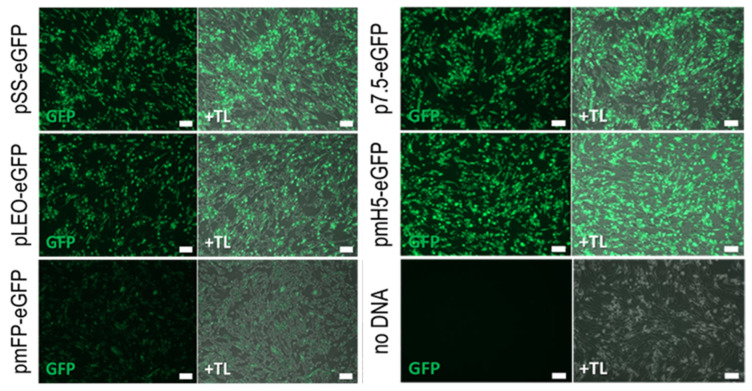
Expression of eGFP from different promoters in LSDV-infected BHK-21 cells. BHK-21 cells were infected with nLSDVSODis-UCT at an MOI of 0.5 and transfected with 2 ug plasmid, encoding the eGFP gene driven by poxvirus promoters pSS, pLEO, pmFP, p7.5 or pmH5. Images were taken two days post infection using green fluorescence (GFP) and phase contrast merged with green fluorescence (+TL). pSS = synthetic vaccinia virus promoter, pLEO = synthetic late-early promoter, pmFP = fowlpox virus promoter modified to remove late promoter element, p7.5 = vaccinia virus 7.5 kDa promoter, pmH5 = modified vaccinia virus H5 promoter. Scale bar = 100 µm.

Poxviruses use specific promoters for expression, with VACV promoters being the best characterised [42]. The activity of previously characterized poxvirus promoters, placed upstream of a GFP reporter gene, were tested in transient expression assays in LSDV-infected cells to determine if they would be recognized by LSDV. All five promoters, namely pSS, pLEO, pmFP, p7.5 and pmH5, were active in combination with LSDV (Figure 1). In the absence of LSDV infection, no eGFP expression was seen from cells transfected with the reporter plasmids.

### 3.2. Construction of Recombinant LSDVGC5 in BHK-21 Cells

**Figure 2:**
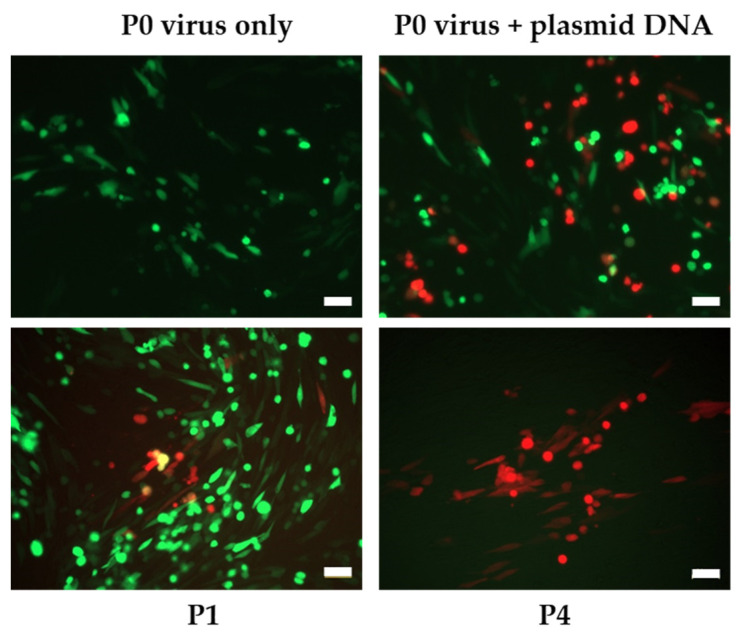
Generation of recombinant LSDVGC5 in BHK-21 cells from the parent LSDV(SODis)BEFV-Gb expressing eGFP. Plasmid DNA = transfer vector containing mCherry marker. P0 = initial infection and transfection of BHK-21 cells, P1 = passage 1, P4 = passage 4. Scale bar = 50µm.

Recombinant LSDV, expressing HIV-1 env and gag genes, together with mCherry as a fluorescent marker, was constructed (Figure 2). Single LSDVGC5 clones were generated in BHK-21 cells by picking mCherry positive foci (passage (P) 1) followed by limited dilution ranges of virus supernatant after freeze thawing (>P1). By P4, wells containing only mCherry positive recombinant LSDV were obtained, thus generating LSDVGC5 (BHK-21).

### 3.3. Evaluation of the Growth of LSDV and Expression of Foreign Genes from LSDV, in BHK Cells, in the Presence and Absence of Sodium Butyrate

**Figure 3:**
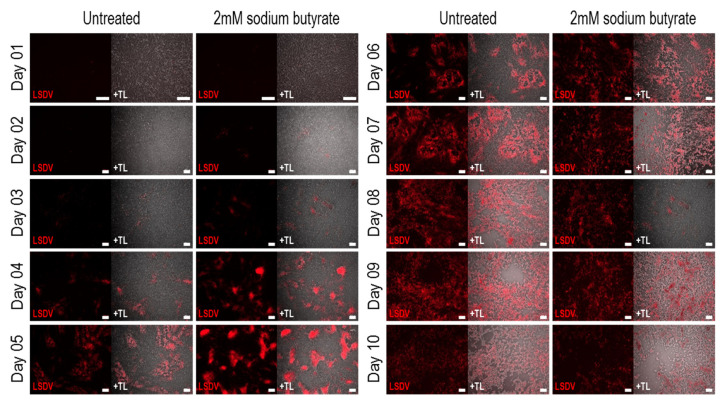
Growth of LSDVGC5 (expressing mCherry) in BHK-21 cells with and without sodium butyrate. Cells were infected on day 0 at MOI = 0.5. Scale bar = 200 µm.

**Figure 4:**
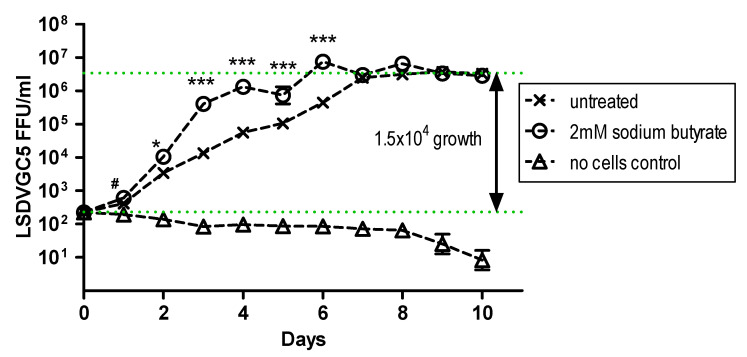
Growth of LSDVGC5 in BHK-21 cells in the presence and absence of sodium butyrate (SB). BHK-21 cells were infected at MOI = 0.5 (*n* = 6). */***: untreated vs. SB (*p* < 0.05 or 0.001 respectively), #: SB vs. no cells (*p* < 0.05), untreated or SB vs. no cells (*p* < 0.001) from day 2 to 10.

Growth of LSDVGC5 in BHK-21 cells was demonstrated, and this was enhanced by the addition of the pan-HDAC inhibitor sodium butyrate from days 3 to 6 post infection (Figure 3 and Figure 4). However, by day 7 the titre was the same, whether the cells were treated with sodium butyrate or not (Figure 4). It was noted that, by this time, many of the cells treated with sodium butyrate had died. 

**Figure 5:**
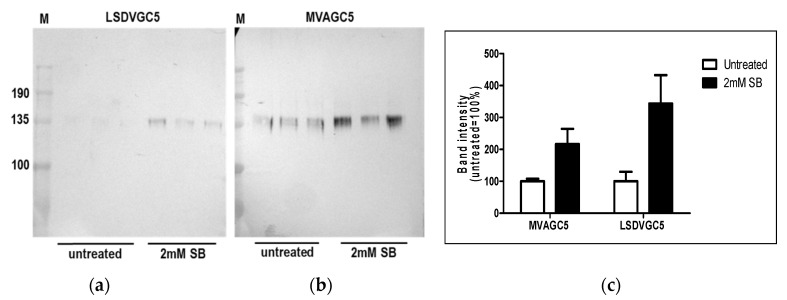
Expression of HIV-1 Env from nonpermissive HEK292T cells infected with LSDVGC5 or MVAGC5 in the presence and absence of 2 mM sodium butyrate. Cell lysates were prepared in triplicate 72 h post infection with LSDVGC5 (**a**) or MVAGC5 (**b**) and subjected to western blot analysis. Densitometry readings were plotted using GraphPad Prism 5 (**c**).

Expression of HIV Env from recombinant poxviruses LSDVGC5 and MVAGC5 was evaluated after the addition of the pan-HDAC inhibitor sodium butyrate. This resulted in increased Env expression as observed by western blot analysis (Figure 5).

### 3.4. The Use of VACV K1L to Improve Growth of LSDV and Select for Recombinant LSDV

**Figure 6:**
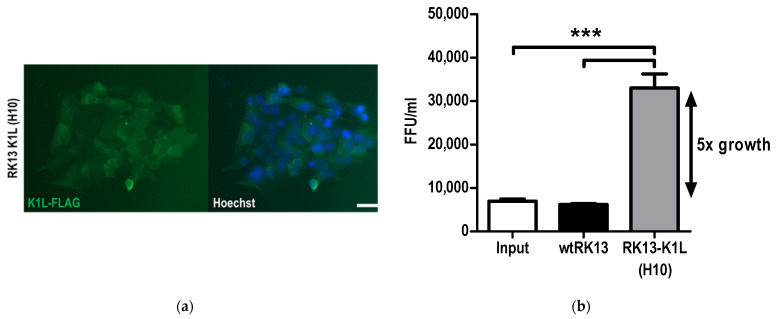
Generation of RK13 cells with stable expression of VACV K1L to rescue growth of LSDV. (**a**) Immunofluorescence showing expression of K1L by the clonal cell lineRK13-K1L(H10); K1L expression was detected by probing for the C-terminal Flag-tag present in the transgene. Scale bar = 100µm. (**b**) Growth of LSDVGC5 in the RK13-K1L(H10) cell line as compared to wildtype RK13 three days post infection. *** *p* < 0.001.

RK13 cells are non-permissive for the growth of LSDV. A stable RK13 cell line, which expressed VACV K1L (RK13-K1L(H10)) was generated (Figure 6a). Comparison of LSDV growth in RK13 (wtRK13) and RK13-K1L(H10) cells showed a five-fold increase in the growth of LSDV in the RK13-K1L(H10) cell line (Figure 6b), confirming that K1L enabled growth of LSDV in RK13 cells. 

**Figure 7:**
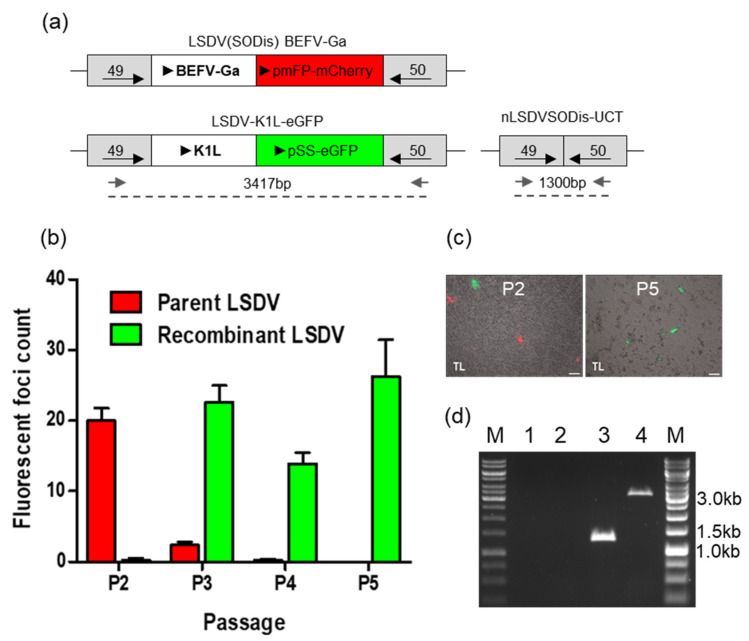
Generation of LSDV- K1L-eGFP in RK13 cells. (**a**) Schematic diagram of the parent virus, LSDV(SODis)BEFV-Ga, LSDV-K1L-eGFP and nLSDVSODis-UCT. (**b**) Fluorescing foci were counted as RK13 cell lysates were passaged (P2 to P5). Red and green bars indicate red and green foci counts respectively. (**c**) Images of passage 2 and 5 of potential LSDV-K1L-eGFP recombinant. Phase contrast with mCherry and eGFP, scale bar = 100µm. (**d**) PCR confirmation of LSDV-K1L-eGFP. DNA was extracted from infected BHK-21 cells and subjected to PCR using forward and reverse primers (small grey arrows) as indicated in A. M—GeneRuler 1 kb DNA Ladder, lane 1—water control, 2—uninfected cells, 3—nLSDVSODis-UCT, 4—LSDV-K1L-eGFP.

To determine whether K1L could be used as a means of selection in the construction of recombinant LSDV, a recombinant LSDV expressing eGFP and K1L was isolated by passage in RK13 cells. Cell lysate was used from LT cells infected with LSDV(SODis)BEFV-Ga and transfected with a transfer vector containing K1L and eGFP between flanking sequences of LSDV ORFs 49 and 50. Figure 7 shows the enrichment of green foci over red foci with passage in RK13 cells. The parent virus, which expressed mCherry, was completely replaced by recombinant LSDV-K1L-eGFP by P5. The recombinant was confirmed to be correct by PCR (Figure 7d) and Sanger sequencing of the gene cassette inserted between LSDV ORFs 49 and 50. This shows that K1L can be used as a means of selection for generating recombinant LSDV. 

There was no growth advantage seen in BHK-21 cells when LSDV-K1L-eGFP was compared to the parent virus (data not shown).

## 4. Discussion

Host-restricted poxviruses are important as vaccine vectors because they have numerous advantages compared to other vaccine vectors. They are heat stable and can be stored at room temperature. Poxviruses have capacity for insertion and expression of up to 25 kbp of foreign DNA compared to adenovirus vectors where package size is limited to 7.5 kbps. They can infect a wide range of cells and express foreign proteins at levels that induce good immune responses. The fact that they replicate in the cytoplasm is another consideration as there is less likelihood of integration into the host genome [2,43,44,45,46]. LSDV has the potential to be added to the list of other poxviruses that have been successfully used in commercial human and veterinary vaccine development [20]. 

It is important to note that poxviruses differ in the distinct types of immune responses induced when used as vaccine vectors. Analysis of the spleen transcriptome to assess the innate immune response in mice to six host restricted poxviruses (lumpy skin disease virus (LSDV), MVA and four Avipoxviruses) demonstrated quantitatively distinct host responses. LSDV, followed by MVA, induced the greatest interferon (IFN) response, while CNPV and fowlpoxvirus induced the up regulation of two immunoglobulin genes (Ighg and Ighg3 (IgG3)) with CNPV inducing a third, Ighm (IgM) [47]. HIV-1-specific IgG3 antibodies have been reported to correlate with decreased risk of HIV-1 infection in the RV144 trial, which included a CNPV-based vector [48]. MVA- and LSDV- vectored HIV-1 vaccines have been shown to give superior T cell responses in heterologous (vs homologous) prime-boost experiments [10] and recombinant LSDV was able to significantly boost recombinant MVA primed responses in rhesus macaques [9]. Although there is evidence that MVA can be used repeatedly as a vaccine vector in the same host [49] it is expected that eventually anti-vector immunity will play a role in suppressing immune responses to boosts based on the same vector. The distinct characteristics of the various poxvirus vectors and improvements in immune responses with heterologous prime boost vaccinations justify further development of more poxvirus vectors, including LSDV.

The demonstration that LSDV can grow to relatively high titres in BHK-21 cells, and the fact that this cell line is suitable for the generation of recombinant LSDV opens the possibility of improved manufacturing processes for both human and veterinary vaccines. Confirmation of a range of VACV promoter activity increases the number of poxvirus promoters that can be used to generate recombinants. While many researchers want increased promoter activity, high-strength promoters can result in an excess of foreign protein leading to instability and selection of unstable recombinants [50]. In poxvirus vaccine design, early promoters that enhance antigen expression also improve the antigen-specific CD8 T-cell responses [51]. Our study showed that LSDV replication can be further enhanced by the addition of the pan-HDAC inhibitor sodium butyrate. Although sodium butyrate improved expression of the recombinant genes in MVA and LSDV, it is not certain whether this is due to increased poxvirus growth alone or an increase in protein expression too. An additional lever on improving foreign gene expression could be of particular interest for recombinants expressing foreign genes which may cause instability during manufacture.

Selection of poxvirus recombinants by homologous recombination is a long and laborious process and so alternatives are needed. Recent improvements have included the synthesis of horsepox virus from chemically synthesized DNA, which could be used to make recombinants [52], and the use of CRISPR/Cas9 technology to target the parent poxvirus genome [53]. Both these approaches are relatively expensive. In our study, we demonstrate that partial LSDV growth was observed in RK13 cells, but only when the VACV host gene K1L was expressed. Despite the limited growth, the expression of K1L was enough to serve as a positive selection marker for the generation of recombinant LSDV vaccines in RK13 cells. The passage of virus in RK13 cells is considerably less laborious than the standard method of picking foci. The simplification of generating (recombinant) LSDV vaccines shown here should increase the interest in this platform for future livestock vaccine development and, with BHK-21 cells approved for current good manufacturing practice, this can be expanded to human vaccines. The inclusion of K1L in LSDV did not result in improved replication in BHK-21 cells but the impact on pathogenicity in vivo, whether in non-permissive or permissive hosts, is not known and remains to be determined. Alternatively, K1L could be removed from the final recombinant, as is often done with MVA, by flanking the K1L gene with repetitive DNA sequences [27]. In MVA, the use of K1L did not enhance replication in human or monkey cell lines [54] or result in replication in rabbits [35]. LSDV is regarded as a very safe vaccine vector for non-permissive hosts as there is no evidence that the virus can replicate in non-ruminant hosts. Therefore, the risk of rescue of a replicating virulent LSDV under these circumstances is negligible. This is not the case for MVA, which, in very rare circumstances, could theoretically recombine with naturally circulating Orthopoxviruses during co-infection [44]. 

In conclusion, we have demonstrated that BHK-21 cells can be used to grow LSDV and to create recombinant viruses. A number of promoters used for foreign gene expression in vaccinia virus have been shown to work for LSDV too. We have also shown that K1L can be used for selection of recombinant LSDV in RK13 cells. These are significant advances in the quest to develop LSDV as a vaccine vector, both for animal and human use.

## Figures and Tables

**Table 1:** Poxvirus promoter sequences.

Promoter	Sequence	Size	Ref
pSS	AAAATTGAAATTTTATTTTTTTTTTTTGGAATATAAATA	39 bp	[36]
pLEO	TTTTATTTTTTTTTTTTGGAATATAAATATCCGGTAAAATTGAAAAAATATACACTAATTAGCGTCTCGTTTCAGACGCTAG	82 bp	[37]
pmFP	AGAAAAATATCCTAAAATTGAATTGTAATTATCGATAATAA	41 bp	[38,39]
p7.5	TCCAAACCCACCCGCTTTTTATAGTAAGTTTTTCACCCATAAATAATAAATACAATAATTAATTTCTCGTAAAAGTAGAAAATATA TTCTAATTTATTGCACGG	104 bp	[40]
pmH5	AAAAATTGAAAATAAATACAAAGGTTCTTGAGGGTTGTGTTAAATTGAAAGCGAGAAATAATCATAAATAA	71 bp	[41]
